# Validity of leptin receptor-deficiency (*db/db*) type 2 diabetes mellitus mice as a model of secondary osteoporosis

**DOI:** 10.1038/srep27745

**Published:** 2016-06-10

**Authors:** Le Huang, Yong-ke You, Tracy Y Zhu, Li-zhen Zheng, Xiao-ru Huang, Hai-yong Chen, Dong Yao, Hui-yao Lan, Ling Qin

**Affiliations:** 1 Musculoskeletal Research Laboratory, Department of Orthopaedics and Traumatology, the Chinese University of Hong Kong, Hong Kong SAR, China; 2 Department of Medicine & Therapeutics, the Chinese University of Hong Kong, Hong Kong SAR, China; 3 Bone Quality and Health Assessment Centre, Department of Orthopaedics and Traumatology, The Chinese University of Hong Kong, Hong Kong SAR, China

## Abstract

This study aimed to evaluate the validation of the leptin receptor-deficient mice model for secondary osteoporosis associated with type 2 diabetes mellitus (T2DM) at bone micro-architectural level. Thirty three 36-week old male mice were divided into four groups: normal control (*db/m*) (n = 7), leptin receptor-deficient T2DM (*db/db*) (n = 8), human C-reactive protein (CRP) transgenic normal control (*crp/db/m*) (n = 7), and human CRP transgenic T2DM (*crp/db/db*) (n = 11). Lumber vertebrae (L5) and bilateral lower limbs were scanned by micro-CT to analyze trabecular and cortical bone quality. Right femora were used for three-point bending to analyze the mechanical properties. Trabecular bone quality at L5 was better in *db/db* or *crp/db/db* group in terms of bone mineral density (BMD), bone volume fraction, connectivity density, trabecular number and separation (all p < 0.05). However the indices measured at proximal tibia showed comparable trabecular BMD and microarchitecture among the four groups. Femur length in *crp/db/db* group was significantly shorter than *db/m* group (p < 0.05) and cortices were thinner in *db/db* and *crp/db/db* groups (p > 0.05). Maximum loading and energy yield in mechanical test were similar among groups while the elastic modulus in *db/db* and *crp/db/db* significantly lower than *db/m*. The leptin-receptor mice is not a proper model for secondary osteoporosis associated with T2DM.

Type 2 diabetes mellitus (DM) is a major metabolic disorder that causes profound medical and socioeconomic burden. It currently affects about 382 million adults worldwide and it is projected to reach 592 million by 2030^1^. Type 2 DM is associated with a series of complications that can affect multiple organ systems, including the bone. A recent meta-analysis of 12 studies shows that adults with type 2 DM are at a heightened risk of hip fracture with a relative risk of 1.7^2^. Paradoxically, areal bone mineral density (BMD) measured by dual-energy X-ray absorptiometry (DXA) is found to be normal or higher in these patients^3^,^4^,^5^, leading to the hypothesis that type 2 DM is associated with material and microstructural deficits of the bones which contribute to the increased fracture risk^6^,^7^.

The underlying mechanism for type 2 DM-induced osteoporosis is still elusive. It may involve excessive accumulation of advance glycosylation end-products as a result of hyperglycemia, increased fat deposition at the bone marrow and impaired osteoblast function^8^,^9^,^10^,^11^,^12^,^13^. C-reactive protein (CRP) is an acute-phase reactant produced primarily in the liver under the stimulation of pro-inflammatory cytokines such as interleukin-6 and tumor necrosis factor-α. Elevated CRP levels represent a state of low grade inflammation which has been shown to disturb bone metabolism and has been shown to be associated with enhanced risk of fragility fracture in the general population^14^,^15^,^16^,^17^. A number of studies have described elevated CRP levels in patients with type 2 DM^18^,^19^,^20^. Whether bone fragility in type 2 DM is a result of elevated CRP levels is yet to be determined.

Leptin receptor-deficient (*db/db*) mouse was reported as an animal model of type 2 DM^21^. These animals had autosomal recessive mutation in the leptin receptor and had features typical of type 2 DM, including hyperphagic, obese and hyperglycemia. The aim of this study was to evaluate bony statues at microarchitectural level to determine whether leptin receptor-deficient mice model was a potentially valid animal model for type 2 DM-associated secondary osteoporosis.

## Materials and Methods

### Groups

Thirty three 36-week old male mice purchased from Laboratory Animal Services Centre of the Chinese University of Hong Kong were used and grouped into 4 groups: normal control mice (*db/m*) (n = 7), leptin receptor knock-out type 2 DM mice (*db/db*) (n = 8), human CRP transgenic normal control mice (*crp/db/m*) (n = 7) and human CRP transgenic *db/db* type 2 DM mice (*crp/db/db*) (n = 11). All animals were sacrificed by injecting excessive pentobarbital after being anesthesia by ketamine solution at 36 weeks. Body weight of all the mice was recorded. Lumbar vertebrae 4 to 6 (L4–6) and bilateral lower limbs were harvested. Right femora were preserved in saline before three-point bending test while the rest of the samples were fixed with formalin and kept in 70% ethanol before use. The study was conducted with the approval from the Animal Experimentation Ethics Committee of the Chinese University of Hong Kong (AEEC No. 09/027/MIS) and was conducted according to the approved guideline.

### Evaluation of bone architecture by micro-CT

For evaluation of trabecular bone density and microarchitecture, vertebral body L5 and a section of 850 μm in length of proximal tibia below the growth plate were scanned with a micro-CT system (Micro CT 40, SCANCO Medical, Switzerland) at a voltage of 70 keV in a spatial resolution of 10 μm for animal experimental studies. Measurements were repeated for each sample and the mean values were used for analysis. Volumetric BMD, ratio of bone volume and total volume (BV/TV), trabecular bone number (Tb.N), trabecular bone thickness (Tb.Th), trabecular bone separation (Tb.Sp) and structural model index (SMI) were collected and analyzed. SMI is an index for determining the plate- or rod-like geometry of trabecular structures. For an ideal plate and rod structure, the SMI value is 0 and 3, respectively. For a structure with both plates and rods of equal thickness the value lies between 0 and 3, depending on the volume ratio of rods and plates^22^. For evaluation of cortical bone architecture, a section of 900 μm of the middle shaft of the right femur which comprised pure cortical bone was scanned with the same micro-CT system with settings as above. The following parameters were collected: the diameter of the cross-section area, thickness of the cortical bone.

### Mechanical test

A material test machine (H25KS, Hounsfield Test Equipment Ltd.UK) with a 25N load cell was used for testing mechanical properties of the femur. The left femur was positioned horizontally with the anterior surface upwards and centered on the supports with 10 mm apart. Load was applied at the mid-shaft constantly with displacement rate of 5 mm/min and directed vertically to mid-shaft with anterior surface upward. After testing to failure, the force-displacement curves were recorded. Also the stress-strain curve was obtained by being normalized by stressed area. This conversion would be calculated automatically by the built-in software QMAT (QMAT Professional; Tinius Olsen, Inc. Horsham, PA, USA). Failure force (N), elastic modulus (E-modulus = stress/strain), energy at yield (integration of the total area under the stress-strain curve) were obtained and analyzed by a built-in software QMAT based on our previous established protocols^23^.

### Statistical analyses

All data were expressed as mean and standard deviation. Between-group differences were analyzed by one-way analysis of variance (ANOVA). All statistical analyses were performed by Statistical Package for the Social Sciences (SPSS, version 17.0, Chicago, IL, US). A p-value less than 0.05 was considered statistically significant.

## Results

### Leptin receptor knock-out type 2 DM leads to higher body weight

**Figure 1:**
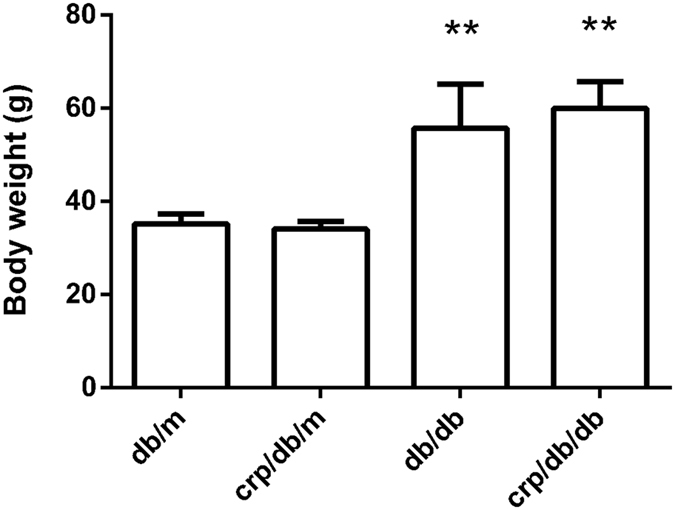
Body weight among the four groups of mice. Data are mean ± SD. **p < 0.05 compared with *db/m* mice.

Human CRP transgene did not affect the body weight. Body weight was comparable between *db/m* and *crp/db/m* mice and between *db/db* and *crp/db/db* mice. Compared with *db/m* mice, *db/db* and *crp/db/db* mice had 58.5% and 70.7%, respectively, significantly higher body weight (both p < 0.05). Compared with *crp/db/m* mice, body weight was also significantly higher in *db/db* and *crp/db/db* mice (both p < 0.05) (Fig. 1).

### Leptin receptor knock-out type 2 DM mice have higher trabecular bone density and better microarchitecture at L5

**Figure 2:**
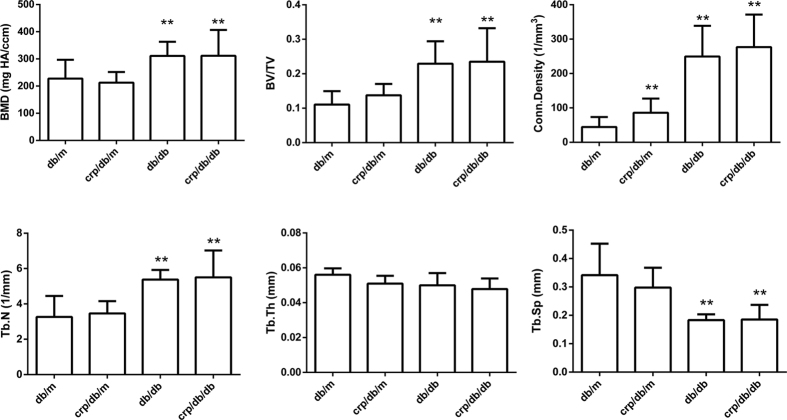
Trabecular bone quality parameters by micro-CT of the L5 vertebra in the four groups of mice. BMD: bone mineral density; Tb.N: trabecular number; Tb.Th: trabecular thickness; Tb.Sp: trabecular separation. Data are mean ± SD. **p < 0.05 compared with *db/m* mice.

**Figure 3:**
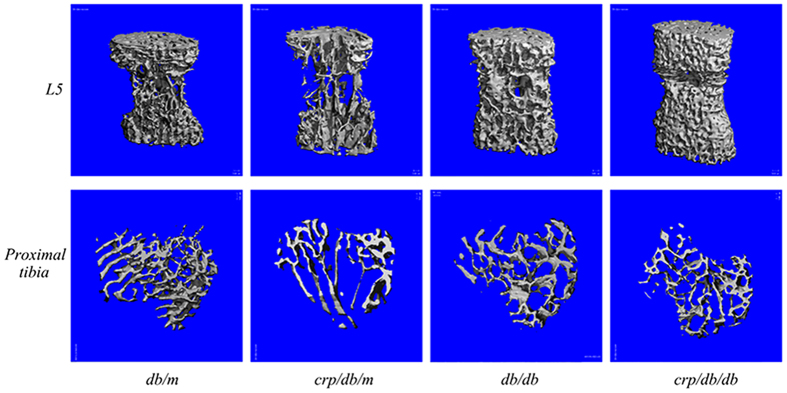
3D images of the trabecular bone by micro-CT of the L5 vertebra (upper row) and proximal tibia (bottom row) in the four groups of mice.

In general, indices of trabecular bone microarchitecture at L5 were comparable between *db/m* and *crp/db/m* mice, except that connectivity density was significantly higher in *crp/db/m* (Fig. 2). Compared to *db/m* mice, BMD, BV/TV, connectivity density, Tb.N and were all significantly higher and Tb.Sp significantly lower in *db/db* or *crp/db/db* mice (all p < 0.05) (Fig. 2). The largest difference was seen in connectivity density, which was 5.6 times and 6.2 times higher for *db/db* and *crp/db/db* mice, respectively. Tb.Th did not differ among the four groups. The average SMI of *db/db* and *crp/db/db* mice was 1.202 and 0.889, respectively, which was lower than those of *db/m* (1.995) or *crp/db/m* (1.671) mice. This indicated that the trabeculae at L5 contained more plate-like structure for *db/db* and *crp/db/db* mice while more rod-like structure for *db/m* and *crp/db/m* mice (Table 1). Indices of trabecular bone were comparable between *db/db* and *crp/db/db* mice. The 3D images of trabecular bone in L5 are shown in Fig. 3 where the images show particularly denser and better connected trabeculae in *db/db* and *crp/db/db* mice compared with *db/m* and *crp/db/m* mice.

### Comparable trabecular bone density and microarchitecture at the proximal tibia among groups

**Figure 4:**
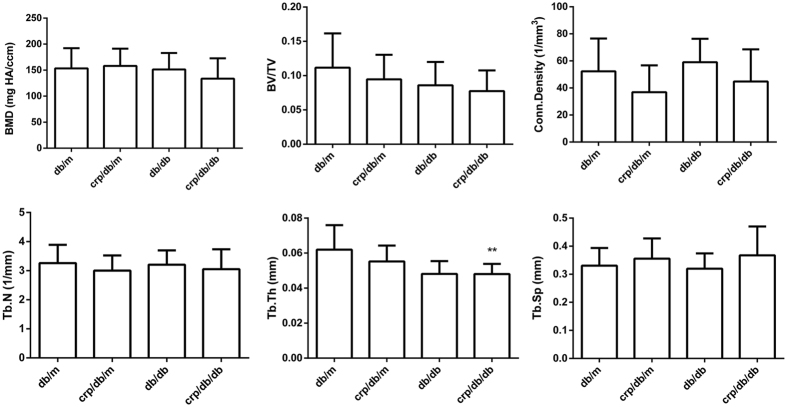
Trabecular bone quality parameters by micro-CT of the proximal tibia in the four groups of mice. BMD: bone mineral density; Tb.N: trabecular number; Tb.Th: trabecular thickness; Tb.Sp: trabecular separation. Data are mean ± SD. **p < 0.05 compared with *db/m* mice.

In contrast to the findings at L5, in general, indices of trabecular bone microarchitecture at the proximal tibia did not differ significantly among the four groups of mice (Fig. 4). BMD was also found comparable among groups. There was a trend towards a lower BV/TV in *db/db* (mean: 0.086) and *crp/db/db* (0.077) mice compared with *db/m* (0.112) and *crp/db/m* (0.095) mice. Connectivity density tended to be lower in *crp/db/m* and *crp/db/db*. However, these differences did not reach statistical significance. Tb.N and Tb.Sp did not differ significantly among groups. Tb.Th was similar between *db/db* and *crp/db/db* mice with average Tb.Th being 0.048 mm for both groups, respectively. Significant difference in Tb.Th was only found between *db/m* and *crp/db/db* mice. SMI at proximal tibia ranged from 2.08 to 2.48, with comparable SMI among groups, representing the trabecular bone at this region tended to contain more rod-like structure (Table 1). Figure 3 shows the 3D image of trabecular bone at the proximal tibia. The images show trabecular bone with similar density and connectivity among the four groups.

### Comparable cortical bone architecture at mid femoral shaft and mechanical properties among groups

**Figure 5:**
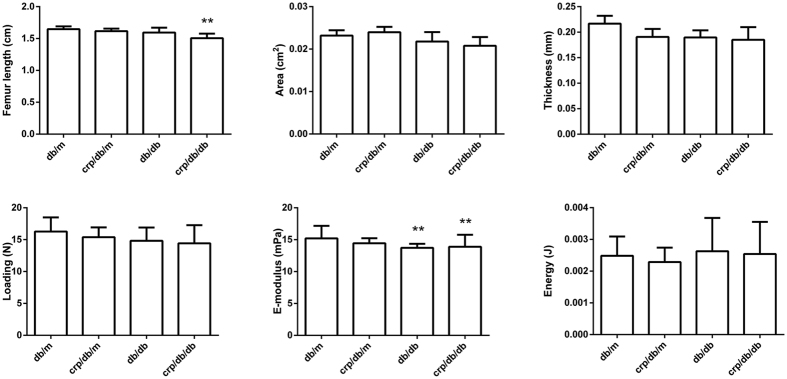
Length, cortical area and thickness, and mechanical properties of the femur among the four groups of mice. E-modulus: elastic modulus. Data are mean ± SD. **p < 0.05 compared with *db/m* mice.

The length of the femur was 8.55% (p < 0.05) significantly shorter in *crp/db/db* mice than in *db/m* mice (Fig. 5). Cross-sectional area and cortical thickness of the femur tended to be lower in *db/db* and *crp/db/db* group compared with *db/m* and *crp/db/m* mice but the differences were not statistically significant (p > 0.05). Group-wise differences in the maximum loading and energy yield were not significantly different (all p > 0.05) (Fig. 5). Compared with *db/m* mice, elastic modulus was 9.75% and 8.67% significantly lower (both p < 0.05) in *db/db* and *crp/db/db* mice, respectively.

## Discussion

In this study, we investigated the bone density and microarchitecture at the spine and limb in a type 2 DM mice model. Leptin receptor knock-out type 2 DM led to significantly higher body weight and human CRP gene did not affect body weight as body weight was comparable between *db/m* and *crp/db/m* mice, and between *db/db* and *crp/db/db* mice. Assessment by micro-CT revealed high bone mass phenotype at L5 in type 2 DM mice (*db/db*) and type 2 DM mice with human CRP transgenic gene (*crp/db/db*), evidenced by significantly higher trabecular BMD, BV/TV, connectivity density, Tb.N and lower Tb.Sp. At the proximal tibia, Tb.Th was significantly lower in *crp/db/db* mice and there was a trend towards lower trabecular BV/TV in *db/db* and *crp/db/db* mice. Otherwise, cortical and trabecular bone density and microarchitecture at the proximal tibia was maintained in type 2 DM mice. Three-point bending test showed significantly lower elastic modulus and comparable loading and energy in type 2 DM mice.

Leptin receptor knock-out type 2 DM mice did not exhibit phenotype, on a bone microarchitectural level, typical of osteoporosis. Despite a large body of clinical and epidemiological studies linking type 2 DM to increased bone fragility and fracture risk, patients with type 2 DM are often found to have higher or normal BMD. Studies investigating bone microarchitecture in animal models of type 2 DM yield conflicting results^24^,^25^,^26^,^27^,^28^,^29^,^30^. The high bone mass phenotype at L5 in *db/db* mice found in our study is in consistent with the study by Ducy *et al*.^24^. They showed significantly higher trabecular BV/TV at L5 and femur in 6-month-old *db/db* mice. This high bone mass phenotype was found in both genders, despite coexisting hypogonadism and hypercortisolism and the appearance of this phenotype preceded the onset of obesity^24^. Higher trabecular bone mass at the spine (L2–3) has also been reported in a leptin-deficient (*ob/ob*) mice model^27^.

In contrast, a number of studies have reported significantly lower bone mass and/or compromised trabecular microarchitecture in *db/db*^25^,^28^,^30^ or *ob/ob* mice model^25^,^27^, or obese Zucker (*fa/fa*) rats model^26^,^29^, which is a type 2 DM model with an inactivating mutation of the leptin receptor. However, the majority of this low bone mass phenotype was found at the limbs (femur or tibia)^25^,^26^,^27^,^28^,^29^ whilst the bone mass at the spine was reported to be normal^25^ or higher^27^. In the study by Williams *et al*., although bone mass reduction was found at both the tibia and spine in *db/db* mice, the magnitude of the decrease was less in the spine evidenced by the finding that significant deterioration of trabecular compartment at L5 was only found in Tb.Th^30^. Our findings are consistent with these previous results in that we found trends towards lower trabecular bone mass and Tb.Th at the tibia in *db/db* mice despite the high bone mass phenotype at the L5. Additionally, bone material property at the femur was also affected in *db/db* mice, evidenced by a lower elastic modulus. These findings suggest that leptin- or leptin receptor-deficiency produces differing bone phenotypes at the spine and limb. The greater loss of bone at the limb than at the spine could be due to the greater muscle loss at the limb as a result of obesity^27^. Bone remodeling is significantly affected by the muscle because muscle contraction produces the greatest load on bone^31^,^32^ and because muscle mass is the primary determinant of blood flow to the limb^33^,^34^. In *ob/ob* mice, the low bone mass at the femur was associated with the relatively low hind limb muscle mass. In addition, Hamrick *et al*. proposed that the difference in bone phenotype between the femur and lumbar spine of *ob/ob* mice could be partly explained by the differing response of marrow cells at these two skeletal sites to leptin deficiency^27^. Leptin deficiency and obesity are known to be associated with increased bone marrow adipogenesis which has been link to bone loss^35^,^36^. Marked adiposity in the femur suggesting excessive bone marrow adipogenesis, along with an absence of increased adipocyte number in the lumbar vertebrae, has been reported in *ob/ob* mice^27^.

CRP is a measure of chronic inflammation and is an independent risk factor for type 2 DM and cardiovascular disease^37^. Elevated CRP level can also interact with other risk factors to accelerate the progression of type 2 DM and its related complications, particularly cardiovascular diseases^37^,^38^. Previous studies have also found significant correlation between elevated CRP level and increased risk of fracture in general population and this correlation is independent of areal BMD measured by DXA^14^,^15^,^16^,^17^. In this study, we included groups of human CRP transgenic mice to investigate the role of CRP in the development of osteoporosis in type 2 DM. Although *crp/db/db* mice had shorter femur length than *db/db* mice, overall, *crp/db/db* mice did not exhibit poorer bone density and architecture than *db/db* mice. These findings do not support that CRP plays a detrimental role in deteriorating bone density and architecture in type 2 DM. However, our study did not examine degree of mineralization or potential micro-damage to bone matrix or properties of non-mineral phase of the bone, on which CRP might have a direct or indirect effect. The exact mechanism how CRP disturb bone metabolism and induces bone fragility in general population or in type 2 DM requires further studies.

There are some limitations of this study. First, due to the difficulty of measuring the femoral neck of mice, we performed the micro-CT scanning on the L5 and the proximal tibia instead. Our results may not be applicable to the femur which is a major site for fracture in type 2 DM. Second, we did not perform assessments on bone turnover markers by blood sample or bone histomorphometry. The underlying alteration in the balance of bone metabolism responsible for the observed bone phenotype in this animal model was not studied. Finally, assessment of bone quality was conducted only at bone microarchitectural level. Some features of bone microarchitecture such as cortical porosity, micro-crack of one matrix or properties of non-mineral phase of the bone were not be able examined due to limitation of the assessment technology used for the current study. However, mechanical test was conducted to assess the whole bone strength and the results did not show significantly reduced bone strength in *db/db* mice.

Due to above mentioned study limitations, further studies are recommended. First of all since this study aimed to validate the secondary osteoporotic animal model, the mechanistic studies on how leptin receptor knock-out would induce increase of body weight would be subject to explore. Since we all know that leptin plays an important role in not only the contribution of type 2 DM but also bone metabolism, how this mutation would affect the bone quality would also be of our interests apart from weight bearing that is a known factor preventing bone loss. Moreover, as mentioned in results, the mineral phase of bone was not affected by the elevation of CRP the non-mineral phrase should be analyzed to understand the role of CRP on the bone structure.

In conclusion, the leptin receptor-deficient (*db/db*) mice did not exhibit significant compromised bone quality at microarchitectural level. However, deficiency in leptin signaling had differing effect on the spine and limb with high bone mass phenotype being particularly noticeable at the spine. There were trends towards lower trabecular bone mass and compromised microarchitecture at the tibia in the *db/db* mice. Overall, this mouse model does not exhibit phenotype typical of secondary osteoporosis. Human CRP transgenic *db/db* mice did not exhibit poorer bone mass and microarchitecture than *db/db* mice, suggesting that CRP does not play a detrimental role in deteriorating bone density and architecture in type 2 DM.

### Ethics Approval.

The study was cond.ucted with the approval from the Animal Experimentation Ethics Committee of the Chinese University of Hong Kong (AEEC No. 09/027/MIS).

## Additional Information

**How to cite this article**: Huang, L. *et al*. Validity of leptin receptor-deficiency (*db/db*) type 2 diabetes mellitus mice as a model of secondary osteoporosis. *Sci. Rep.* **6**, 27745; doi: 10.1038/srep27745 (2016).

## Figures and Tables

**Table 1:** Structural Model Index (SMI) of the microarchitecture of trabecular bone at L5 vertebra and proximal tibia*.

Groups	L5 vertebra	Proximal tibiae
*db/m*	2.00 ± 0.81	2.49 ± 0.84
*crp/db/m*	1.67 ± 0.61	2.08 ± 0.64
*db/db*	1.20 ± 1.16	2.41 ± 0.92
*crp/db/db*	0.89 ± 1.67	2.27 ± 0.52

^*^SMI is an index for determining the plate- or rod-like geometry of trabecular structures. For an ideal plate and rod structure, the SMI value is 0 and 3, respectively. For a structure with both plates and rods of equal thickness the value lies between 0 and 3, depending on the volume ratio of rods and plates^22^.
